# About the polymorphism of [Li(C_4_H_8_O)_3_]I: crystal structures of trigonal and tetra­gonal polymorphs

**DOI:** 10.1107/S160053681402529X

**Published:** 2014-11-21

**Authors:** Stefanie Gärtner, Tobias Gärtner, Ruth-Maria Gschwind, Nikolaus Korber

**Affiliations:** a University of Regensburg, Institute of Inorganic Chemistry, Universitätsstrasse 31, 93053 Regensburg, Germany; b University of Regensburg, Institute of Organic Chemistry, Universitätsstrasse 31, 93053 Regensburg, Germany

**Keywords:** crystal structure, polymorphism, THF solvate, lithium complexes

## Abstract

Two new trigonal and tetra­gonal polymorphs of the title compound, iodido­tris­(tetra­hydro­furan-κ*O*)lithium, are presented, which both include the isolated ion pair Li(THF)_3_^+^·I^−^. One Li—I ion contact and three tetra­hydro­furan (THF) mol­ecules complete the tetra­hedral coordination of the lithium cation. The three-dimensional arrangement in the two polymorphs differs notably. In the trigonal structure, the ion pair is located on a threefold rotation axis of space group *P*-3 and only one THF mol­ecule is present in the asymmetric unit. In the crystal, strands of ion pairs parallel to [001] are observed with an eclipsed conformation of the THF mol­ecules relative to the Li⋯I axis of two adjacent ion pairs. In contrast, the tetra­gonal polymorph shows a much larger unit cell in which all atoms are located on general positions of the space group *I*4_1_ *cd*. The resulting three-dimensional arrangement shows helical chains of ion pairs parallel to [001]. Apart from van der Waals contacts, no remarkable inter­molecular forces are present between the isolated ion pairs in both structures.

## Abstract 2

Two new polymorphs of the ion pair LiI(C_4_H_8_O)_3_ (trigonal, space group *P* 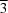; tetra­gonal, space group *I*4_1_ *cd*) show different three-dimensional arrangements in the crystal structure and co-exist at the same temperature.

## Chemical context   

The tetra­hedral arrangement of the [Li(THF)_3_]^+^·I^−^ ion pair has already been reported in the monoclinic crystal structure (space group *P*2_1_/*n*) by Nöth & Waldhör (1998 ▶). Crystals of this phase could be obtained during the reaction of tmp_2_AlI (tmp = tetra­methyl­piperidine) with LiHAsPh (Ph = phen­yl) in toluene/tetra­hydro­furan (THF) or, more conveniently, from LiH and iodine in THF. The applied crystallization temperature was 233 K and the data collection for structure analysis was performed at 193 K. 
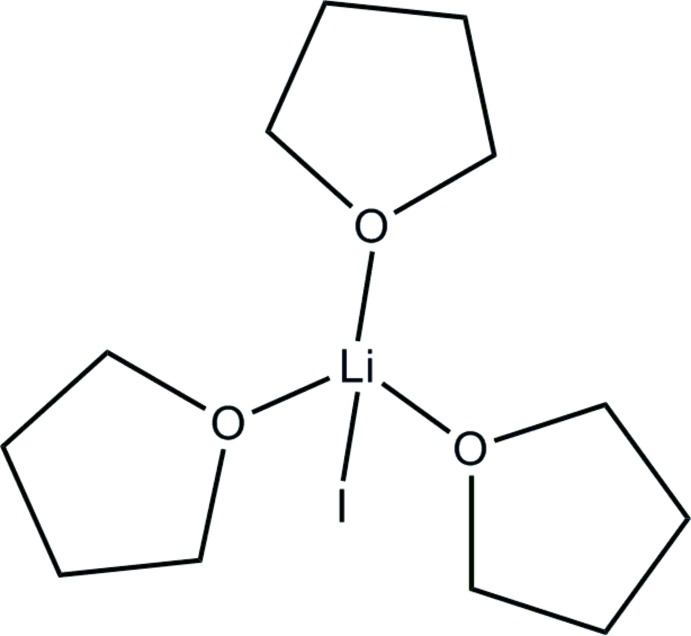


**Figure 1:**
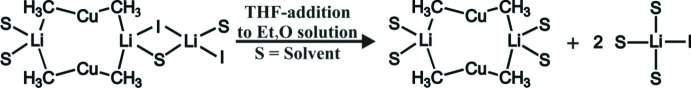
Proposed by NMR in solution: THF addition to iodidocuprates in diethyl ether solutions yields predominantly iodine-free cuprates and solvated Li–I units.

In our case, we obtained two new polymorphs of [Li(THF)_3_]^+^·I^−^ from a solution of (H_3_C)_2_CuLi·LiI in diethyl ether covered with THF. The reaction mixture was stored at 193 K, and the measurements for the single-crystal structure analysis were performed at 123 K. The observation of such contact ion pairs directly confirms the NMR spectroscopic findings (Henze *et al.*, 2005 ▶) that upon addition of THF, the LiI units are separated from the cuprate by the coordination of Li^+^ by three THF mol­ecules (Fig. 1 ▶).

## Structural commentary   

The polymorphs reported herein are higher in symmetry compared to the known monoclinic phase as they crystallize in the trigonal space group *P* 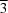 and the tetra­gonal space group *I*4_1_ *cd*. In the asymmetric unit of the trigonal polymorph, the lithium and iodide ion pair is located on a threefold rotation axis (Wyckoff position 2*d*) and one THF mol­ecule is located on a general position. This results in a symmetric coordination of the lithium cation by the three THF mol­ecules. The unit cell of this polymorph is small and contains two formula units. In contrast, in the structure of the tetra­gonal polymorph, all atoms are located on general positions. The resultant unit cell is considerably larger and contains 16 formula units. Nevertheless, the mol­ecular structures of the [Li(THF)_3_]^+^·I^−^ ion pair in all three polymorphs are very similar in terms of bond lengths and angles. Table 1 ▶ compiles Li—I and Li—O distances for all three structures.

## Supra­molecular features   

The reasons for the same mol­ecular [Li(THF)_3_]^+^·I^−^ unit crystallizing in three different crystal systems and space groups lies in the supra­molecular assembly of these ion pairs. The three-dimensional arrangement of the [Li(THF)_3_]^+^·I^−^ ion pairs is different in all three known polymorphs. The differences in the supra­molecular structures can best be demonstrated when taking the shortest supra­molecular Li⋯I distances (∼5.7 Å) into account. Although this is a formal procedure since at distances of more than 5 Å no chemically reasonable inter­actions are present, it allows for a better understanding of the packing of the ion pairs in the unit cell.

**Figure 2:**
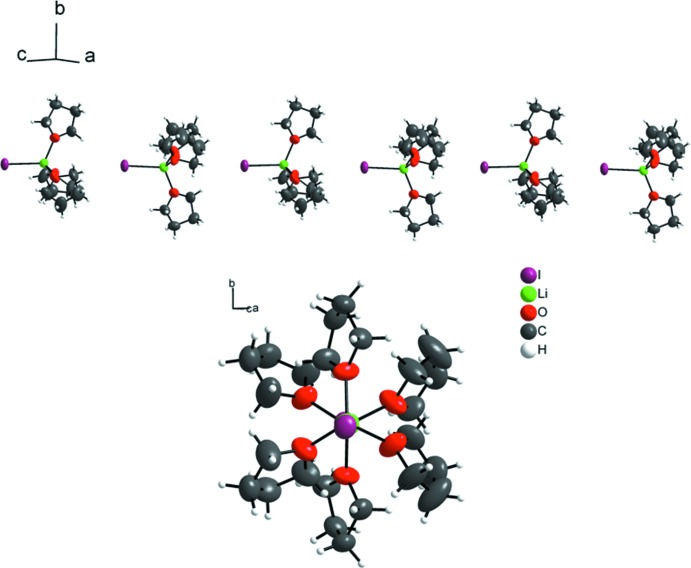
Linear chains in the monoclinic polymorph of [Li(THF)_3_]^+^·I^−^ (top) show a staggered arrangement of the THF mol­ecules relative to the Li⋯I axis (bottom). Displacement ellipsoids (except for hydrogen atoms) are drawn at the 50% probability level.

In the previously reported monoclinic structure, the formation of linear chains of individual ion pairs parallel to [10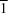] is observed (Fig. 2 ▶, top), where the THF mol­ecules form a staggered conformation relative to a fictive Li—I axis of the shortest supra­molecular Li⋯I distance (Fig. 2 ▶, bottom). The complete structure is characterized by anti­parallel oriented chains. The resulting calculated density of the compound is 1.468 g cm^−3^ (Nöth & Waldhör, 1998 ▶).

**Figure 3:**
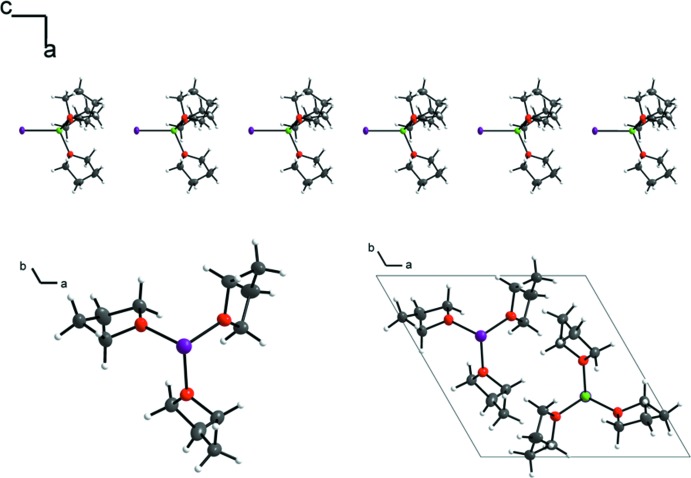
Linear chains extend parallel to [001] in the trigonal polymorph (top) and show an eclipsed conformation of the THF mol­ecules relative to the Li⋯I axis (bottom, left) in an anti­parallel arrangement in the unit cell (bottom, right). Displacement ellipsoids (except for hydrogen atoms) are drawn at the 50% probability level.

A similar supra­molecular arrangement is found in the trigonal structure. Here, the ion pairs are likewise aligned in linear chains, in this case parallel to [001] (Fig. 3 ▶, top), but in contrast to the monoclinic variant, the THF mol­ecules assemble in an eclipsed fashion relative to the fictive Li—I axis of the shortest supra­molecular Li⋯I distance (Fig. 3 ▶, bottom left). These chains again are packed with an anti­parallel orientation in the crystal structure (Fig. 3 ▶, bottom right), and the calculated density is 1.516 g cm^−3^.

**Figure 4:**
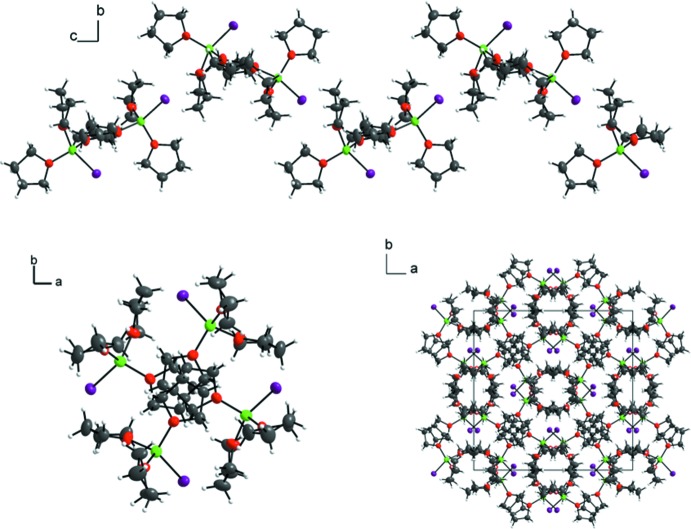
Helical chains parallel to [001] (top and bottom, left) are present in the crystal structure of the tetra­gonal polymorph. Displacement ellipsoids (except for hydrogen atoms) are drawn at the 50% probability level.

Finally, in the tetra­gonal structure, the situation is completely different, as the ion pairs form helical chains along the 4_1_ screw axis of space group *I*4_1_ *cd* (Fig. 4 ▶, top and bottom left). This assembly in the unit cell (Fig. 4 ▶, bottom right) results in a calculated density of 1.503 g cm^−3^.

The higher temperature during synthesis/crystallization of the monoclinic polymorph compared to the conditions applied for the title compounds obviously caused the crystallization of the two new polymorphs. Both have a very similar density and co-exist in one reaction batch. At higher temperatures, the crystals became amorphous, indicating an irreversible phase transition.

## Synthesis and crystallization   

A Schlenk flask, equipped with a stirring bar and 0.5 mmol (1 eq) CuI, was dried four times *in vacuo* to remove residual moisture. Then 5 ml of diethyl ether was added and the Cu(I) salt was suspended. Upon addition of 2 eq (H_3_C)Li in diethyl ether, the mixture gave a colourless solution. After removal of the stirring bar, the solution was covered with THF. The flask was then stored at 193 K. After several days, clear colourless needles could be observed. Suitable crystals were isolated in nitro­gen-cooled perfluoro­ether oil and mounted on the goniometer for data collection at 123 K. The crystals of the two compounds did not differ in their forms. For several crystals, the unit cell was determined, proving the presence of either the tetra­gonal or the trigonal polymorph.

## Refinement   

Crystal data, data collection and structure refinement details are summarized in Table 2 ▶. The positions of the lithium cations were located in difference Fourier maps. H atoms were positioned with idealized geometry and were refined with C—H = 0.99 Å and *U*_iso_(H) = 1.2*U*_eq_(C).

## Supplementary Material

Crystal structure: contains datablock(s) LiI_3THF_trigonal, LiI_3THF_tetragonal. DOI: 10.1107/S160053681402529X/wm5091sup1.cif

Structure factors: contains datablock(s) LiI_3THF_trigonal. DOI: 10.1107/S160053681402529X/wm5091LiI_3THF_trigonalsup2.hkl

Structure factors: contains datablock(s) LiI_3THF_tetragonal. DOI: 10.1107/S160053681402529X/wm5091LiI_3THF_tetragonalsup3.hkl

CCDC references: 1034840, 1034841

Additional supporting information:  crystallographic information; 3D view; checkCIF report

## Figures and Tables

**Table 1:** LiI and LiO distances () of the [Li(THF)_3_]I unit in all three known polymorphs. Data for the monoclinic polymorph are from Nth Waldhr (1998 ▶).

	monoclinic	trigonal	tetragonal
LiI	2.741(7)	2.744(7)	2.721(11)
LiO1	1.927(7)	1.932(4)	1.934(13)
LiO2	1.915(8)		1.961(13)
LiO3	1.947(7)		1.944(14)

**Table 2:** Experimental details

	Trigonal polymorph	Tetragonal polymorph
Crystal data		
Chemical formula	[Li(C_4_H_8_O)_3_]I	[Li(C_4_H_8_O)_3_]I
*M*_r_	350.15	350.15
Crystal system, space group	Trigonal, *P* 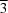	Tetragonal, *I*4_1_ *c* *d*
Temperature (K)	123	123
*a*, *b*, *c* ()	10.2530(14), 10.2530(14), 8.4250(17)	18.288(3), 18.288(3), 18.511(4)
, , ()	90, 90, 120	90, 90, 90
*V* (^3^)	767.0(3)	6191(2)
*Z*	2	16
Radiation type	Mo *K*	Mo *K*
(mm^1^)	2.08	2.06
Crystal size (mm)	0.10 0.07 0.05	0.10 0.05 0.03

Data collection		
Diffractometer	Stoe IPDS	Stoe IPDS
Absorption correction	Analytical (*X-RED* and *X-SHAPE*; Stoe Cie, 2002 ▶)	Analytical (*X-RED* and *X-SHAPE*; Stoe Cie, 2002 ▶)
*T*_min_, *T*_max_	0.760, 0.827	0.629, 0.744
No. of measured, independent and observed [*I* > 2(*I*)] reflections	4938, 1185, 994	14474, 2802, 2130
*R*_int_	0.048	0.044
(sin /)_max_ (^1^)	0.652	0.605

Refinement		
*R*[*F* ^2^ > 2(*F* ^2^)], *wR*(*F* ^2^), *S*	0.029, 0.067, 1.01	0.027, 0.058, 0.95
No. of reflections	1185	2802
No. of parameters	52	154
No. of restraints	0	1
H-atom treatment	H-atom parameters constrained	H-atom parameters constrained
_max_, _min_ (e ^3^)	1.26, 0.35	0.74, 0.21
Absolute structure		Flack *x* determined using 922 quotients [(*I* ^+^)(*I* )]/[(*I* ^+^)+(*I* )] (Parsons *et al.*, 2013 ▶)
Absolute structure parameter		0.03(2)

Computer programs: *X-AREA* (Stoe Cie, 2002 ▶), *SHELXS97* and *SHELXL2014* (Sheldrick, 2008 ▶), *DIAMOND* (Brandenburg, 2012 ▶) and *OLEX2* (Dolomanov *et al.*, 2009 ▶).

## supplementary crystallographic information

### Crystal data

**Table:**

[Li(C_4_H_8_O)_3_]I	*D*_x_ = 1.503 Mg m^−^^3^
*M**_r_* = 350.15	Mo *K*α radiation, λ = 0.71073 Å
Tetragonal, *I*4_1_*c**d*	θ = 2.2–25.5°
*a* = 18.288 (3) Å	µ = 2.06 mm^−^^1^
*c* = 18.511 (4) Å	*T* = 123 K
*V* = 6191 (2) Å^3^	Needle, clear colourless
*Z* = 16	0.10 × 0.05 × 0.03 mm
*F*(000) = 2816	

### Data collection

**Table:**

Stoe IPDS diffractometer	2130 reflections with *I* > 2σ(*I*)
Graphite monochromator	*R*_int_ = 0.044
ω scans	θ_max_ = 25.5°, θ_min_ = 2.2°
Absorption correction: analytical (*X-RED* and *X-SHAPE*; Stoe & Cie, 2002)	*h* = −21→22
*T*_min_ = 0.629, *T*_max_ = 0.744	*k* = −22→21
14474 measured reflections	*l* = −20→22
2802 independent reflections	

### Refinement

**Table:**

Refinement on *F*^2^	Hydrogen site location: inferred from neighbouring sites
Least-squares matrix: full	H-atom parameters constrained
*R*[*F*^2^ > 2σ(*F*^2^)] = 0.027	*w* = 1/[σ^2^(*F*_o_^2^) + (0.0296*P*)^2^] where *P* = (*F*_o_^2^ + 2*F*_c_^2^)/3
*wR*(*F*^2^) = 0.058	(Δ/σ)_max_ = 0.001
*S* = 0.95	Δρ_max_ = 0.74 e Å^−^^3^
2802 reflections	Δρ_min_ = −0.21 e Å^−^^3^
154 parameters	Absolute structure: Flack *x* determined using 922 quotients [(*I*^+^)-(*I*^-^)]/[(*I*^+^)+(*I*^-^)] (Parsons *et al.*, 2013)
1 restraint	Absolute structure parameter: −0.03 (2)
Primary atom site location: structure-invariant direct methods	

### Special details

**Table:**

Experimental. crystal mounting in perfluorether (T. Kottke, D. Stalke, J. Appl. Crystallogr. 26, 1993, p. 615)
Geometry. All e.s.d.'s (except the e.s.d. in the dihedral angle between two l.s. planes) are estimated using the full covariance matrix. The cell e.s.d.'s are taken into account individually in the estimation of e.s.d.'s in distances, angles and torsion angles; correlations between e.s.d.'s in cell parameters are only used when they are defined by crystal symmetry. An approximate (isotropic) treatment of cell e.s.d.'s is used for estimating e.s.d.'s involving l.s. planes.
Refinement. Refinement of *F*^2^ against ALL reflections. The weighted *R*-factor *wR* and goodness of fit *S* are based on *F*^2^, conventional *R*-factors *R* are based on *F*, with *F* set to zero for negative *F*^2^. The threshold expression of *F*^2^ > σ(*F*^2^) is used only for calculating *R*-factors(gt) *etc*. and is not relevant to the choice of reflections for refinement. *R*-factors based on *F*^2^ are statistically about twice as large as those based on *F*, and *R*- factors based on ALL data will be even larger.

### Fractional atomic coordinates and isotropic or equivalent isotropic displacement parameters (Å^2^)

**Table:**

	*x*	*y*	*z*	*U*_iso_*/*U*_eq_	
I1	0.52335 (2)	0.24851 (5)	0.75879 (6)	0.04154 (13)	
C4	0.3881 (8)	0.4855 (7)	0.6892 (7)	0.048 (3)	
H4A	0.3499	0.4836	0.6514	0.058*	
H4B	0.4341	0.5032	0.6671	0.058*	
O1	0.3985 (3)	0.4151 (3)	0.7204 (3)	0.0466 (14)	
C1	0.3853 (6)	0.4187 (5)	0.7953 (5)	0.057 (2)	
H1A	0.4234	0.3913	0.8220	0.069*	
H1B	0.3370	0.3971	0.8069	0.069*	
C2	0.3867 (10)	0.4949 (9)	0.8153 (8)	0.065 (4)	
H2A	0.4364	0.5095	0.8311	0.078*	
H2B	0.3519	0.5046	0.8552	0.078*	
C3	0.3650 (6)	0.5353 (5)	0.7492 (7)	0.065 (3)	
H3A	0.3906	0.5829	0.7461	0.077*	
H3B	0.3116	0.5438	0.7481	0.077*	
O2	0.3480 (3)	0.2781 (3)	0.6374 (3)	0.0416 (14)	
C9	0.4427 (5)	0.3790 (6)	0.5212 (6)	0.061 (3)	
H9A	0.4138	0.4247	0.5247	0.073*	
H9B	0.4090	0.3376	0.5127	0.073*	
C12	0.5599 (4)	0.3560 (5)	0.5662 (5)	0.052 (2)	
H12A	0.5796	0.3126	0.5915	0.063*	
H12B	0.5897	0.3991	0.5795	0.063*	
O3	0.4851 (4)	0.3676 (4)	0.5859 (4)	0.0423 (18)	
C10	0.4998 (11)	0.3845 (10)	0.4602 (8)	0.074 (5)	
H10A	0.4815	0.3616	0.4152	0.089*	
H10B	0.5127	0.4361	0.4502	0.089*	
C11	0.5620 (5)	0.3447 (5)	0.4885 (5)	0.057 (2)	
H11A	0.6082	0.3639	0.4680	0.069*	
H11B	0.5581	0.2921	0.4768	0.069*	
C7	0.2604 (5)	0.1901 (5)	0.6642 (5)	0.045 (2)	
H7A	0.2555	0.1929	0.7174	0.053*	
H7B	0.2413	0.1424	0.6473	0.053*	
C6	0.2217 (5)	0.2522 (6)	0.6282 (5)	0.051 (3)	
H6A	0.1756	0.2645	0.6535	0.061*	
H6B	0.2109	0.2411	0.5769	0.061*	
C8	0.3374 (4)	0.2008 (4)	0.6414 (4)	0.0432 (17)	
H8A	0.3713	0.1788	0.6770	0.052*	
H8B	0.3462	0.1778	0.5937	0.052*	
C5	0.2781 (5)	0.3140 (5)	0.6351 (7)	0.055 (3)	
H5A	0.2751	0.3475	0.5932	0.066*	
H5B	0.2697	0.3425	0.6799	0.066*	
Li1	0.4357 (6)	0.3295 (6)	0.6710 (6)	0.039 (3)	

### Atomic displacement parameters (Å^2^)

**Table:**

	*U*^11^	*U*^22^	*U*^33^	*U*^12^	*U*^13^	*U*^23^
I1	0.03936 (19)	0.0441 (2)	0.0412 (2)	0.0038 (4)	−0.0042 (6)	0.00914 (17)
C4	0.055 (7)	0.044 (6)	0.046 (7)	0.006 (5)	0.000 (5)	0.002 (5)
O1	0.067 (4)	0.041 (3)	0.031 (3)	0.015 (3)	−0.001 (3)	0.002 (2)
C1	0.075 (6)	0.057 (6)	0.041 (6)	0.018 (4)	0.008 (4)	0.006 (4)
C2	0.088 (11)	0.062 (9)	0.045 (9)	0.010 (8)	−0.003 (8)	−0.004 (6)
C3	0.092 (7)	0.042 (5)	0.059 (7)	0.014 (4)	−0.003 (6)	0.003 (5)
O2	0.037 (3)	0.039 (3)	0.049 (4)	0.004 (2)	0.000 (3)	0.005 (2)
C9	0.049 (5)	0.085 (7)	0.049 (8)	0.009 (4)	−0.009 (5)	0.015 (6)
C12	0.034 (4)	0.070 (6)	0.053 (6)	0.001 (4)	0.009 (4)	0.008 (4)
O3	0.035 (3)	0.058 (4)	0.034 (4)	0.010 (3)	−0.001 (3)	0.009 (3)
C10	0.077 (9)	0.106 (11)	0.039 (9)	0.010 (10)	0.009 (7)	0.009 (8)
C11	0.052 (6)	0.066 (6)	0.054 (7)	−0.007 (4)	0.012 (4)	−0.006 (4)
C7	0.041 (4)	0.043 (5)	0.050 (6)	−0.006 (4)	0.006 (4)	−0.009 (4)
C6	0.033 (4)	0.056 (5)	0.063 (7)	0.002 (5)	0.007 (5)	−0.008 (5)
C8	0.049 (4)	0.038 (4)	0.043 (5)	0.002 (3)	0.001 (3)	−0.001 (3)
C5	0.034 (4)	0.053 (6)	0.080 (7)	0.005 (4)	0.001 (4)	0.014 (7)
Li1	0.047 (7)	0.040 (6)	0.031 (7)	0.003 (5)	−0.002 (5)	0.002 (5)

### Geometric parameters (Å, º)

**Table:**

I1—Li1	2.721 (11)	C12—H12A	0.9900
C4—H4A	0.9900	C12—H12B	0.9900
C4—H4B	0.9900	C12—O3	1.432 (11)
C4—O1	1.425 (13)	C12—C11	1.453 (14)
C4—C3	1.496 (16)	O3—Li1	1.944 (14)
O1—C1	1.410 (10)	C10—H10A	0.9900
O1—Li1	1.934 (13)	C10—H10B	0.9900
C1—H1A	0.9900	C10—C11	1.448 (19)
C1—H1B	0.9900	C11—H11A	0.9900
C1—C2	1.442 (19)	C11—H11B	0.9900
C2—H2A	0.9900	C7—H7A	0.9900
C2—H2B	0.9900	C7—H7B	0.9900
C2—C3	1.484 (18)	C7—C6	1.496 (15)
C3—H3A	0.9900	C7—C8	1.483 (11)
C3—H3B	0.9900	C6—H6A	0.9900
O2—C8	1.429 (9)	C6—H6B	0.9900
O2—C5	1.438 (10)	C6—C5	1.535 (14)
O2—Li1	1.961 (13)	C8—H8A	0.9900
C9—H9A	0.9900	C8—H8B	0.9900
C9—H9B	0.9900	C5—H5A	0.9900
C9—O3	1.442 (13)	C5—H5B	0.9900
C9—C10	1.542 (18)		
			
H4A—C4—H4B	108.6	C12—O3—Li1	126.7 (7)
O1—C4—H4A	110.4	C9—C10—H10A	111.1
O1—C4—H4B	110.4	C9—C10—H10B	111.1
O1—C4—C3	106.7 (9)	H10A—C10—H10B	109.0
C3—C4—H4A	110.4	C11—C10—C9	103.5 (10)
C3—C4—H4B	110.4	C11—C10—H10A	111.1
C4—O1—Li1	126.0 (7)	C11—C10—H10B	111.1
C1—O1—C4	109.4 (8)	C12—C11—H11A	110.7
C1—O1—Li1	124.3 (6)	C12—C11—H11B	110.7
O1—C1—H1A	110.3	C10—C11—C12	105.4 (9)
O1—C1—H1B	110.3	C10—C11—H11A	110.7
O1—C1—C2	107.2 (10)	C10—C11—H11B	110.7
H1A—C1—H1B	108.5	H11A—C11—H11B	108.8
C2—C1—H1A	110.3	H7A—C7—H7B	109.1
C2—C1—H1B	110.3	C6—C7—H7A	111.2
C1—C2—H2A	110.7	C6—C7—H7B	111.2
C1—C2—H2B	110.7	C8—C7—H7A	111.2
C1—C2—C3	105.3 (11)	C8—C7—H7B	111.2
H2A—C2—H2B	108.8	C8—C7—C6	102.9 (7)
C3—C2—H2A	110.7	C7—C6—H6A	111.4
C3—C2—H2B	110.7	C7—C6—H6B	111.4
C4—C3—H3A	111.0	C7—C6—C5	101.8 (7)
C4—C3—H3B	111.0	H6A—C6—H6B	109.3
C2—C3—C4	103.6 (9)	C5—C6—H6A	111.4
C2—C3—H3A	111.0	C5—C6—H6B	111.4
C2—C3—H3B	111.0	O2—C8—C7	105.9 (6)
H3A—C3—H3B	109.0	O2—C8—H8A	110.6
C8—O2—C5	109.5 (6)	O2—C8—H8B	110.6
C8—O2—Li1	124.7 (6)	C7—C8—H8A	110.6
C5—O2—Li1	121.2 (7)	C7—C8—H8B	110.6
H9A—C9—H9B	108.9	H8A—C8—H8B	108.7
O3—C9—H9A	110.8	O2—C5—C6	105.2 (6)
O3—C9—H9B	110.8	O2—C5—H5A	110.7
O3—C9—C10	104.7 (10)	O2—C5—H5B	110.7
C10—C9—H9A	110.8	C6—C5—H5A	110.7
C10—C9—H9B	110.8	C6—C5—H5B	110.7
H12A—C12—H12B	108.5	H5A—C5—H5B	108.8
O3—C12—H12A	110.2	O1—Li1—I1	111.4 (5)
O3—C12—H12B	110.2	O1—Li1—O2	104.5 (6)
O3—C12—C11	107.4 (8)	O1—Li1—O3	104.8 (6)
C11—C12—H12A	110.2	O2—Li1—I1	114.2 (5)
C11—C12—H12B	110.2	O3—Li1—I1	113.9 (5)
C9—O3—Li1	118.4 (7)	O3—Li1—O2	107.1 (6)
C12—O3—C9	108.8 (8)		
			
C4—O1—C1—C2	−16.1 (14)	C11—C12—O3—C9	−11.9 (10)
O1—C4—C3—C2	17.6 (12)	C11—C12—O3—Li1	139.8 (9)
O1—C1—C2—C3	27.1 (14)	C7—C6—C5—O2	27.9 (10)
C1—C2—C3—C4	−27.1 (12)	C6—C7—C8—O2	34.6 (9)
C3—C4—O1—C1	−1.5 (13)	C8—O2—C5—C6	−7.1 (9)
C3—C4—O1—Li1	−175.0 (8)	C8—C7—C6—C5	−37.6 (9)
C9—C10—C11—C12	−31.8 (14)	C5—O2—C8—C7	−17.1 (9)
O3—C9—C10—C11	24.6 (14)	Li1—O1—C1—C2	157.6 (10)
O3—C12—C11—C10	28.1 (12)	Li1—O2—C8—C7	138.7 (7)
C10—C9—O3—C12	−7.9 (12)	Li1—O2—C5—C6	−163.8 (8)
C10—C9—O3—Li1	−162.2 (10)		
